# Supplementary material for: Cancer Associated PRDM9: Implications for Linking Genomic Instability and Meiotic Recombination
Source: Int J Mol Sci. 2023 Nov 20;24(22):16522. doi: 10.3390/ijms242216522 (PMC10671843; doi:10.3390/ijms242216522)
Supplement: Supplementary file 1 [file ijms-24-16522-s001.zip › Supplementary dataset 2.pdf]

1. Algorithm in R for detecting PRDM9 motifs in breakpoint regions

```
library(BSgenome.Hsapiens.UCSC.hg19.masked)
genome <- BSgenome.Hsapiens.UCSC.hg19.masked
seqnames <- seqnames(genome)
prdm9table <- read.table("C:/Work/prdm9.txt", header =
TRUE)

for (i in 7:1024)
{

    for (seqname in seqnames)
    {
        x <- genome[[seqname]]


        (paste("Run PRDM9 no. ", seqname, i, prdm9table$PRDM9[i]))
        Lpattern <- toString(prdm9table$PRDM9[i])
        M1 <- matchPattern(Lpattern, x, max.mismatch=0, min.mismatch=0,
with.indels=FALSE, fixed=TRUE, algorithm="auto")
        #print(paste("M1 = ", M1))
        myfile <- paste("C:\\Work\\GeneScreening\\prdm9\\",
prdm9table$PRDM9[i], ".bed", sep="")
        sink(myfile, append=TRUE)
        if (length(M1) != 0)
        {
            chrvalue <- seqname
            for (k in 1:length(M1))
            {
                cat(chrvalue)
                cat("\t")
                cat(start(M1[k]))
                cat("\t")
                cat(end(M1[k]))
                cat("\t")
                cat(toString(prdm9table$PRDM9[i]))
                cat("\n")
            }
        }
        sink()


    }

    myfile2 <- paste("C:\\Work\\GeneScreening\\prdm9\\lz",
prdm9table$PRDM9[i], ".jpg", sep="")
    myfile3 <- paste("C:\\Work\\GeneScreening\\prdm9\\pt",
prdm9table$PRDM9[i], ".jpg", sep="")
    myfile4 <- paste("C:\\Work\\GeneScreening\\prdm9\\output",
prdm9table$PRDM9[i], ".txt", sep="")
library("regioneR")
    test <- import(myfile)
    test
```

```
 OV500_path <- system.file("tests", package = "rtracklayer")
  OV500_bed <- file.path(OV500_path, "OV500.bed")
  OV500 <- import(OV500_bed)
 OV500
  pt <- overlapPermTest(A=OV500, B=test, ntimes=700)
  pt
  lz <- localZScore(pt=pt, A=OV500, B=test)
  jpeg(myfile2)
  plot(lz)
  dev.off()
  jpeg(myfile3)
  plot(pt)
  dev.off()
  output <- overlapRegions(test, OV500)
  output
  write.table(output, myfile4, append = FALSE, quote = TRUE, sep = " ",
      eol = "\n", na = "NA", dec = ".", row.names = TRUE,
      col.names = TRUE, qmethod = c("escape", "double"),
      fileEncoding = "")



}
```

2. Script in C# for detecting all possible variations of PRDM9 degenerate sequence motif

```
#include <stdio.h>
#include <stdlib,h>
#include <string.h>
int main(int argc *argv[])
{

   char num[10];
            num[0]= 'A';
            num[1]= 'G';
            num[2]= 'T':
            num[3]= 'C';
            int i,j,k,l,m;
            int count=0;
            for (i = 0; i <= 3; i++){
               for (j = 0; j <=3; j++) {
                        for(k = 0; k <= 3; k++) {
                                    for ( l = 0; l <= 3; l++) {
                                                for (m = 0; m <= 3; m++) {
                                                         printf("CC%cCC%cT%c%cCC%cC \n", num[i], num[j],
num[k], num[l], num[m]
                                                             );
                                                count++;
```

```
                                                    }
                                                      }
                                                     }
                                                    }
        }
        printf("total combinations %d ", count);
        return 0;
}
```
