# Supplementary material for: Cancer Associated PRDM9: Implications for Linking Genomic Instability and Meiotic Recombination
Source: Int J Mol Sci. 2023 Nov 20;24(22):16522. doi: 10.3390/ijms242216522 (PMC10671843; doi:10.3390/ijms242216522)
Supplement: Supplementary file 1 [file ijms-24-16522-s001.zip › Supplemntary file 1/Flow chart 1.pdf]

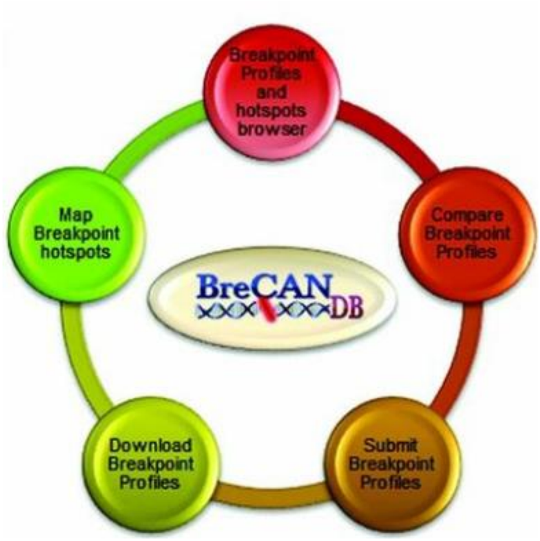

DSBs locally stored, flanking frames are produced

Use of R's **matchPattern** function

A text file is produced with all the hits containing the PRDM9 pattern

We use R's **regioneR** library in order to perform R's **overlapPermTest** function

We use R's **regioneR** library in order to perform R's **localZScore** function

We store **overlapPermTest** and **localZScore** plots in .jpg picture files

We use R's **regioneR** library in order to perform R's **overlapRegions** function

Finally, we store the output of **overlapRegions** function to a text file
